# The effect of sodium butyrate on the growth characteristics of human cervix tumour cells.

**DOI:** 10.1038/bjc.1992.172

**Published:** 1992-06

**Authors:** J. E. Dyson, J. Daniel, C. R. Surrey

**Affiliations:** Academic Unit of Radiotherapy, Cookridge Hospital, Leeds, UK.

## Abstract

Sodium butyrate has been shown to affect cell proliferation, and, at concentrations above approximately 0.5 mM, to cause cell death in some tumour cell lines. When combined with cytotoxic drugs increase in chemosensitivity has been observed. We are presently carrying out a study of the combined effects of sodium butyrate and cytotoxic drugs on cultured cervix tumour cells. To provide a baseline for this study we have carried out a systematic investigation of the effects of sodium butyrate alone on the growth characteristics of cervix tumour cells cultured as multicell spheroids. This has shown that concentrations of n-butyrate of 0.005 mM to 0.50 mM decrease cell proliferation without inducing cell death, the effect increasing with increasing concentration. Butyrate concentrations greater than 0.50 mM cause cell death after a period of 5 to 15 days exposure, dependent on concentration. Concentrations of 0.010 mM and above cause fragmentation of, and increased cell shedding from, multicell spheroids, suggesting an effect on the cell surface. Concentrations of butyrate greater than 0.10 mM cause a considerable increase in the synthesis of cytokeratin, as shown by reaction with cytokeratin antibody. Correlated with this is a marked increase in cell size, concentrations of butyrate of 2.0 or 3.0 mM leading to an approximate doubling of cell diameter, followed by cell disintegration. The effects of butyrate less than 0.25 mM are readily reversible. At concentrations greater than 0.25 mM the effects are reversible up to a limit of about 7 to 20 days depending on concentration, even when cytokeratin synthesis has been induced.


Br. J. Cancer (1992), 65, 803–808 © Macmillan Press Ltd., 1992

### The effect of sodium butyrate on the growth characteristics of human cervix tumour cells

J.E.D. Dyson[1,2] J. Daniel[5] & C.R. Surrey[1]

[1]*Academic Unit of Radiotherapy and* [2]*Department of Radiobiology, Tunbridge Building, Cookridge Hospital, Leeds LS16 6QB, UK.*

**Summary** Sodium butyrate has been shown to affect cell proliferation, and, at concentrations above approximately 0.5 mM, to cause cell death in some tumour cell lines. When combined with cytotoxic drugs increase in chemosensitivity has been observed. We are presently carrying out a study of the combined effects of sodium butyrate and cytotoxic drugs on cultured cervix tumour cells. To provide a baseline for this study we have carried out a systematic investigation of the effects of sodium butyrate alone on the growth characteristics of cervix tumour cells cultured as multicell spheroids. This has shown that concentrations of n-butyrate of 0.005 mM to 0.50 mM decrease cell proliferation without inducing cell death, the effect increasing with increasing concentration. Butyrate concentrations >0.50 mM cause cell death after a period of 5 to 15 days exposure, dependent on concentration. Concentrations of 0.010 mM and above cause fragmentation of, and increased cell shedding from, multicell spheroids, suggesting an effect on the cell surface. Concentrations of butyrate >0.10 mM cause a considerable increase in the synthesis of cytokeratin, as shown by reaction with cytokeratin antibody. Correlated with this is a marked increase in cell size, concentrations of butyrate of 2.0 or 3.0 mM leading to an approximate doubling of cell diameter, followed by cell disintegration. The effects of butyrate <0.25 mM are readily reversible. At concentrations >0.25 mM the effects are reversible up to a limit of about 7 to 20 days depending on concentration, even when cytokeratin synthesis has been induced.

Although early-stage cervical cancer is generally associated with a good prognosis, disease free survival rates for advanced disease patients are poor. *In vitro* studies of cervical cancer cells have shown cervical cancer to be relatively chemoresistant disease (Welander & Parker, 1987). In recurrent disease prior radiotherapy or surgery may affect subsequent use of chemotherapy by interference with access of the drug(s) to malignant cells (e.g. Connors, 1984). Possibly because of these, and other factors, response of cervical cancer to cytotoxic drugs tends to be short lived and incomplete, although cisplatin can provide short term benefit in some patients (Connors, 1984; Sabir *et al.*, 1989; Alberts *et al.*, 1991). However, cytotoxic drugs in conjunction with radiotherapy (Khoury *et al.*, 1991), or combined with agent(s) which act on cells by a different mechanism, may prove more beneficial. Of the latter a group of agents which induce tumour cell maturation or differentiation is currently of much interest (Cheson *et al.*, 1986) either used alone or in combination with cytotoxic drugs or irradiation. Sodium n-butyrate is a member of this group of agents.

The four-carbon fatty acid n-butyrate has been shown to have diverse effects on cell morphology and metabolism *in vitro*, to cause inhibition of cell proliferation, and in some cell lines, and at butyrate concentrations of 0.5 mM and above, to cause differentiation followed by cell death (see e.g. Prasad, 1980; Kruh, 1982; Nordenberg *et al.*, 1987). Possibly combined with the effect of n-butyrate on cell proliferation is its action in reversibly inducing what has been termed a 'better differentiated phenotype', of increased radiosensitivity and chemosensitivity (Spremuli & Dexter, 1984). Due to these properties n-butyrate has been the subject of clinical investigations in leukaemic patients (Novogrodsky *et al.*, 1983; Miller *et al.*, 1987). Its effects when combined with cytotoxic drugs (Wasserman *et al.*, 1989) or irradiation (Arundel & Leith, 1987; Leith *et al.*, 1986) have also been investigated.

We are presently carrying out an investigation of the effect of n-butyrate, when combined with a range of cytotoxic drugs, on cervix tumour cell lines when cultured as multicell spheroids. As a necessary preliminary to this study we have carried out a systematic investigation of the effect of n-butyrate alone on the growth characteristics of cervix tumour cells cultured as multicell spheroids. The effect of n-butyrate concentrations in the range 0.005 to 3 mM has been investigated, and the culture of the cells as multicell spheroids has allowed measurements over periods of up to 24 days. The results of this study are presented in this report.

Correspondence: J.E.D. Dyson, Tunbridge Building, Cookridge Hospital, Leeds LS16 6QB, UK.
Received 8 November 1991; and in revised form 28 January 1992.

### Materials and methods

### *Cell culture*

The cervix tumour cell lines employed in this study were established in primary culture from cervix biopsy tissue taken routinely during radiotherapy at Cookridge Hospital (Dyson *et al.*, 1984*a*). Six cell lines were used: 754, 612, 995, 090, 329, 708. These were maintained as monolayer cultures from which multicellular aggregates were obtained as required by using the method of Sutherland and Durand (1976). Spheroid cultures were initiated with the same number of spheroids per flask to allow intercomparison of cell counts during the period of the experiments. Multicell spheroids were maintained in culture as previously described (Boothby *et al.*, 1989). Excess spheroids were discarded at the time of media change to maintain cell numbers approximately constant.

The necessary volumes of 50 mM or 500 mM sodium n-butyrate solution were added to the spheroid suspensions at the start of the experiment to adjust to the concentrations shown in the figures, with further additions at media changes to maintain these concentrations. The butyrate solution was prepared from n-butyric acid (BDH Limited, Poole, UK) in Hanks basic salt solution, adjusted to pH 7.2 with NaOH solution, then sterilised by filtration through an 0.2 μm Acrodisc (Gelman Sciences Limited, Northampton, UK).

### *Spheroid diameter*

This was measured by means of a laser diffraction particle sizer (Malvern Instruments Limited, Malvern, UK) as previously described (Boothby *et al.*, 1989). The 600 mm range lens was employed covering the range of diameters of 11.6 to 1128 μm.

### Cell numbers

Twenty-five ml aliquots were taken from the stirring flasks and the spheroids and cells collected by centrifugation, the supernatent was discarded and the pellet suspended in 7.5 mM EDTA to disaggregate the spheroids. After 30 min incubation at 37°C the EDTA was removed by centrifugation and the pellet suspended in 1 to 3 ml of phosphate buffered saline (PBS) and vortexed to complete the disaggregation. Triplicate counts were made employing a haemacytometer (improved Neubauer). A record was kept of the volume of excess spheroids discarded, and aliquots removed for measurements, and cell counts were corrected for cumulative volume of spheroids and cells removed.

### Cell size

Spheroid aliquots were disaggregated with EDTA as described above, then examined under the microscope to ensure a single cell suspension had been obtained. Cell size was measured with the laser diffraction particle sizer, with the 100 mm range lens covering the range of diameters of 1.9 to 188 μm.

### Cytokeratin antibody reaction

The spheroid aliquot was disaggregated as described above. The final pellet was cooled in ice, then 4 ml of ice-cold 70% alcohol was added. The fixed cell aliquots were stored at 4°C until processed. The cells were recovered from 70% alcohol by centrifugation then suspended in 1 ml of PBS plus 0.1% Tween 20 (Sigma Chemical Company Limited, Poole, UK), centrifuged again, then the pellet resuspended in 50 μl of PBS/Tween. The antibody used was mouse anti-human cytokeratin which reacts with cytokeratin proteins 8 and 18 (CAM 5.2, Becton Dickinson UK Limited, Oxford, UK), 50 μl was added, mixed thoroughly, then incubated for 30 min at 37°C. A further 1 ml of PBS/Tween was then added, mixed, and the suspension centrifuged and the supernatent removed. The second antibody was then added, 25 μl of goat anti-mouse IgG, whole molecule, conjugated with fluorescein isothiocyanate (FITC) (Sigma F2012, absorbed human serum proteins), 50 μl of PBS/Tween added and mixed and the suspension incubated at 37°C for 30 min. The suspension was centrifuged and the supernatent removed, the pellet was suspended in 0.5 ml PBS then 1 ml of propidium iodide (PI) (Sigma) in PBS added and mixed to give a final concentration of 25 μM PI. The suspension was stored overnight in the refrigerator. The cells were anlaysed with an Ortho Cytofluorograf Systems 50H flow cytometer equipped with a Spectra-Physics 2025-03 argon ion laser operating at the 488 nm line at 400 mW. The green fluorescence intensity, 530 to 565 nm (FITC-cytokeratin) and red fluorescence intensity, >630 nm (PI-DNA) were determined for individual cells and the data stored in an Ortho 2150 computer module interfaced to the systems 50H, for later analysis. The mean value of the green fluorescence intensity distribution was determined by computer analysis. The extent of the increase in cytokeratin content due to cell growth in the presence of n-butyrate was expressed as the ratio of the mean value of the green fluorescence intensity distribution for butyrate grown cells to that of the mean value for control cells, for each concentration of butyrate.

### Ki67 antibody reaction

The antibody used was mouse anti-human Ki67 (M722, Dakopatts Limited, High Wycombe, UK). The processing of the cells was the same as for the cytokeratin reaction, and the same second antibody was used. The extent of the increase in the Ki67 reaction was quantitated and expressed in the same manner as for the cytokeratin reaction.

### Cell cycle analysis

Spheroid aliquots were removed for analysis at designated intervals, disaggregated to a single cell suspension as described above, then prepared and stained with PI and FITC for flow cytometric analysis as previously described (Dyson et al., 1987). Calculation of percent of cells in phases of the cell cycle was carried out from the DNA content profile by means of a cell cycle analysis programme developed by Watson et al. (1987).

### Cell viability

This was assessed by differential permeability to the stains ethidium bromide and acridine orange, determined by flow cytometric measurement (Dyson et al., 1984b).

### Results

The majority of the measurements in this investigation were carried out with two cervix tumour cell lines, 754 and 612. Both cell lines gave concordant results. Four other cervix cell lines, 995, 090, 329, and 708 were used in the measurements of the effect of n-butyrate on cell proliferation rates. These four cell lines gave an essentially similar response pattern to n-butyrate as cell lines 754 and 612. The results presented in this report are those for cervix tumour cell line 754 only to allow a direct intercomparison of the various cell parameters.

The effect on cell proliferation of continuous exposure to concentrations of n-butyrate from 0.005 mM to 1.0 mM is shown in Figure 1 for the period 7 to 24 days from initial

**Figure 1** Reduction in cell proliferation during culture in the presence of the indicated concentrations of n-butyrate. Cell number vs days of exposure to n-butyrate (semi-logarithmic plot). Culture procedure and method of measurement described in Materials and methods. (●) control, no n-butyrate; (◑) 0.005 mM; (∇) 0.010 mM; (▼) 0.025 mM; (■) 0.050 mM; (◐) 0.10 mM; (Δ) 0.25 mM; (□) 0.50 mM; (●) 1.0 mM n-butyrate. Each series of points is the average of four experiments. Bars show standard error of the mean (±s.e.). Slopes to points fitted by eye. Cervix tumour cell line 754.

exposure to n-butyrate. It will be noted that cell numbers for the control give a straight line on the semi-logarithmic plot indicating that culture conditions were such that exponential cell proliferation was maintained. Reductions in cell proliferation may therefore be attributed to the effect of the n-butyrate and not to nutrient deprivation. At the lowest concentration of n-butyrate studies, 0.005 mM, there was, within experimental error, no effect on proliferation rate, although a trend towards a reduction was observed (Figure 1). At 0.01 mM n-butyrate an approximately 50% reduction was observed, increase in cell numbers remained exponential, however, as it did at 0.025 mM n-butyrate (Figure 1). When the n-butyrate concentration was increased to 0.05 mM and above the rate of cell proliferation was observed to decrease further with time of exposure after a period of about 15 to 17 days (Figure 1). At concentrations of 2.0 to 3.0 mM n-butyrate cell proliferation ceased altogether within a period of 1 to 3 days, and then cell death ensured over a period of 7 to 20 days (data not shown).

In addition to the effect on cell proliferation a further effect of n-butyrate on growth of cells as multicell spheroids was observed when increase in spheroid diameter with time was measured (Figure 2). Fragmentation of the spheroids occurred, together with increased cell shedding, this becoming more marked, and occurring at an earlier time, as the concentration of n-butyrate was increased (Figure 2). The particle frequency *vs* spheroid diameter plots displayed by the particle sizer depict this phenomenon (inset to Figure 2). At the highest concentrations of n-butyrate, 1.0 to 3.0 mM, no increase in the diameter of the spheroids was observed and although some cell proliferation occurred at 1.0 mM (Figure 1), none was apparent at 2.0 and 3.0 mM n-butyrate. At 2.0 and 3.0 mM n-butyrate inadequate numbers of cells and cell aggregates remained for measurement after 13 days (Figure 2), although dying cells and much cell debris were observed on microscopic examination.

The highest concentrations of n-butyrate (0.25 mM to 3.0 mM) caused considerable increases in cell diameter (Figure 3). At concentrations of 0.25 and 0.50 mM n-butyrate the measurements suggest that the cells adapted to the presence of the n-butyrate, since after reaching a maximum midway through the measurements the cells had returned to control value by 24 days (Figure 3). At 1.0 mM n-butyrate

**Figure 2** Changes in average spheroid diameter during culture in the presence of the indicated concentrations of n-butyrate. Results of a typical experiment. Culture procedure and method of spheroid measurement described in Materials and methods. (●) control, no n-butyrate; (▽) 0.010 mM; (▼) 0.025 mM; (■) 0.050 mM; (◐) 0.10 mM; (△) 0.25 mM; (□) 0.50 mM; (●) 1.0 mM; (○) 2.0 mM; (▲) 3.0 mM, n-butyrate. Cell line 754. Inadequate numbers of spheroids for measurement remained after day 13 at 2.0 mM and 3.0 mM n-butyrate. Inset to Figure 2 frequency of distribution of spheroid diameters at day 20 for (———) control; (– – –) 0.1 mM; (------) 1.0 mM n-butyrate.

**Figure 3** Changes in average cell diameter during culture in the presence of the indicated concentrations of n-butyrate. Culture procedure and method of measurement of cell diameter described in Materials and methods. (●) control, no n-butyrate; (△) 0.25 mM; (□) 0.50 mM; (●) 1.0 mM; (○) 2.0 mM; (▲) 3.0 mM, n-butyrate. Inadequate numbers of cells at 3.0 mM n-butyrate remained for measurement after day 21. Each series of points is the average of four experiments. Bars show ± s.e. Cell line 754.

the cells appeared to reach a state of equilibrium with little change after 7 days (Figure 3). At 2.0 and 3.0 mM n-butyrate the cells had approximately doubled their diameter by 12 to 17 days; the decrease observed thereafter was most probably due to death and disintegration of the largest cells (Figure 3).

Essentially the same pattern as for increase in cell size was observed for increase in cytokeratin content (Figure 4). A concentration of 0.1 mM n-butyrate produced a trend towards increased cytokeratin in the latter part of the measurements, but within experimental error this was not distinguishable from the control (Figure 4). At days 20 and 24 a significant increase was observed at 0.25 mM n-butyrate (Figure 4). The small amount of cell proliferation permitted by 0.50 and 1.0 mM n-butyrate (Figure 1) possibly allowed a degree of equilibrium to be set up, with cells differentiating and being lost, then being replaced to some extent by cell proliferation. This would explain the equilibrium state suggested by the measurements for these concentrations (Figure 4).

The reversibility of the action of n-butyrate in inducing cytokeratin synthesis is depicted in Figure 5a. The period of exposure to n-butyrate was confined to a period of 7 days, since beyond that few cells remained viable at n-butyrate concentrations of 2 or 3 mM. The effect of n-butyrate on the Ki67 antigen was also measured (Gerdes *et al.*, 1991), during exposure and following its removal, with the intention of employing the anticipated decrease in Ki67 levels as a measure of the reduction in cell proliferation (Gerdes *et al.*, 1984), for comparison with the increase in cytokeratin levels employed as a measure of cell differentiation. We found however, a marked transient increase in the Ki67 antigen, especially at concentrations of 2.0 and 3.0 mM (Figure 5b). The Ki67 antigen level was not related to extent of cell proliferation under these conditions, therefore, but was apparently accumulated, at least initially, due to the presence of butyrate (Figure 5b). The decrease in Ki67 observed after day 4 may have been due to death of those cells with the highest Ki67 content. On removal of the n-butyrate an increase in the Ki67 antigen was observed for previous exposure to 1.0 to 3.0 mM, with a considerable increase after exposure to 3.0 mM (Figure 5b). By 10 days from removal of the n-butyrate Ki67 values had returned nearly to control levels (Figure 5b), as had values for cytokeratin content (Figure 5a).

Exposure to n-butyrate did not cause a major accumula-

Figure 4 Changes in cytokeratin content of cells during culture in the presence of the indicated concentrations of n-butyrate, as measured by extent of reaction with cytokeratin antibody. Culture procedure and method of measurement described in Materials and methods. (◐) 0.10 mM; (△) 0.25 mM; (□) 0.50 mM; (●) 1.0 mM; (○) 2.0 mM; (▲) 3.0 mM, n-butyrate. Inadequate numbers of cells at 3.0 mM n-butyrate remained for measurement after day 17. Each series of points is the average of four experiments. Bars show ± s.e. Cell line 754.

Figure 5 Changes in average cell content of cytokeratin **a**, and Ki67 antigen **b**, during 7 days exposure to n-butyrate and following its removal. Culture procedure and methods of measurement described in Materials and methods. (△) 0.25 mM; (□) 0.50 mM; (●) 1.0 mM; (○) 2.0 mM; (▲) 3.0 mM, n-butyrate. Each series of points is the average of four experiments. Bars show ± s.e. Cell line 754.

tion of cells in any phase of the cell cycle, the only phase in which an appreciable change was observed was $G_2$ where an increase was apparent between 1 and 2 days after initial exposure (Figure 6*c*). Quite marked changes, however, were apparent on removel of the n-butyrate. There was a considerable decrease in the number of cells in $G_1$ as cells moved out of that phase and into S and $G_2$ phases (Figure 6). This was especially marked for passage of cells from $G_1$ to S following exposure to 3.0 mM n-butyrate, and it is interesting that this cell movement paralleled the considerable increase in Ki67 antigen following removal of 3.0 mM n-butyrate (cf Figures 6a, b and 5b).

### Discussion

Culture of the cell lines employed in this investigation as multicell spheroids has allowed observations of the effect of n-butyrate on the cell growth characteristics of cultured cervix tumour cells over the relatively prolonged period of 24 days. Throughout this period the cells were maintained in exponential growth (apart from the effect of n-butyrate, cf Figure 1) by discarding excess cells at media change. This prolonged period of exposure is important since at concentrations of n-butyrate of 0.25 mM and below the effect of n-butyrate on cell proliferation takes some 7 to 10 days to become manifest (cf Figures 1 and 2). It is only at concentrations of 0.50 mM and above that n-butyrate causes cell death in cervix tumour cells, and only at concentrations of 2.0 and 3.0 mM does this appear to be significant. At n-butyrate concentrations of 0.5 mM and above a proportion of the cells, increasing with increasing n-butyrate concentration, synthesise cytokeratin (Figure 4), increase in volume (Figure 3) and eventually disintegrate. On microscopic examination, or flow cytometric analysis, very few dying or dead cells are found in the spheroid cultures, but considerable amounts of cell debris are apparent, principally at 2.0 and 3.0 mM n-butyrate. At n-butyrate concentrations of 0.25 mM and below no cell debris is detectable and the effect of n-butyrate appears to be solely on cell proliferation without causing differentiation and evenutal death, at least within the 24 day period of the measurements.

In addition to the reduction in cell proliferation, or possibily related to this reduction, n-butyrate apparently exerts a

Figure 6 Changes in cell cycle phase fractions during 7 days exposure of cervix tumour cells to n-butyrate, and following its removal. Measurements carried out and values calculated as described in Materials and methods. (●) 1.0 mM; (○) 2.0 mM; (▲) 3.0 mM, n-butyrate. Values shown are the average of three experiments. Bars show ± s.e. Points on extreme left, and horizontal dotted lines, show average control values (± s.e.) during the course of the measurements. Cell line 754.

surface effect on cells cultured in its presence. As shown in Figure 2 n-butyrate caused increased cell shedding from the spheroid surface and spheroids reached an equilibrium volume with cell loss to the medium at a smaller diameter. At intermediate concentrations the spheroids fragment after a period of exposure dependent on the n-butyrate concentration (Figure 2 and inset to Figure 2). Comparison of Figures 1 and 2 shows that this is not due to cell loss or a change in cell proliferation. These results seem to be in conflict with earlier investigations of the effect of n-butyrate which resulted in increased cell adhesion (McGarvey *et al.*, 1990) and increased intercellular adhesion (Frankfurt, 1982). These investigators employed monolayer cultures in their studies; in contrast we commenced our measurements with cell aggregates of approximately 120 $\mu$m diameter, with exposure to lower concentrations of n-butyrate for a more prolonged period. However, the fact that increased adhesion has been observed to be induced by n-butyrate under certain conditions (McGarvey *et al.*, 1990; Frankfurt, 1982) does agree with our observations insofar as it also suggests an effect of n-butyrate on the cell surface. How n-butyrate might modify the cell surface is not at present known, but there is experimental evidence which shows how this could take place. It has proved possible to modify the cell membrane fatty acid composition by modification of the culture medium (see e.g. Spector & Burns, 1987); this also increased cell sensitivity to adriamycin (Spector & Burns, 1987). Stearic acid, an 18-carbon fatty acid, has also been shown to inhibit the growth of human cervical cells (Gleeson *et al.*, 1990). It is, perhaps, unlikely that a 4-carbon fatty such as n-butyrate would become directly incorporated into the cell membrane, it is, however, conceivable that it could modify the action of certain enzymes involved in membrane synthesis and/or influence cellular levels of such enzymes. Butyrate has been shown to modify the activity of various membrane bound enzymes (e.g. Nordenberg *et al.*, 1987; Wasserman *et al.*, 1989).

Attempts have been made, with limited success, to use butyrate alone in the treatment of leukaemia (Novogradsky *et al.*, 1983; Miller *et al.*, 1987). An advantage of butyrate is that it is a naturally occurring substance with little or no apparent toxicity (Miller *et al.*, 1987; Daniel *et al.*, 1989). Set against this, however, is the problem of maintaining effective plasma concentrations in patients, as the rapid metabolism and excretion of n-butyrate necessitates the continuous infusion of large volumes of butyrate solution (Miller *et al.*, 1987). Miller *et al.* (1987) were able to maintain a butyrate concentration of about 0.04 to 0.06 mM for 10 days. Our results for cervix tumour cells show that after 24 days 0.05 mM n-butyrate *in vitro* reduced cell proliferation to 6% of the control value, and the proliferation rate was still decreasing at that time (Figure 1). An increase in n-butyrate concentration to 0.10 mM reduced the proliferation to 2.6% of control at 24 days (Figure 1). Thus it would be advantageous to increase the period of butyrate infusion beyond 10 days and also increase the butyrate plasma concentration if either of these factors can be achieved. Whether this could be done without unacceptable damage to actively dividing normal tissues is not at present known. Possibly a more promising approach is the combination of butyrate infusion with radiotherapy or chemotherapy, which we are presently investigating employing cervix tumour cell lines cultured as multicell spheroids. The effect of the combined treatments on normal tissue morbidity is largely unknown, but there would be the advantage in the case of butyrate combined with radiotherapy that the effect of the combination would largely be restricted to the volume of the tumour. An alternative approach both for the butyrate alone or for combination with other therapy, is the use of derivatives of butyrate which are less readily metabolised (Planchon *et al.*, 1991).

This work was supported by the Yorkshire Cancer Research Campaign, Harrogate, HG1 5LQ, UK, and by the Cookridge Hospital Research Fund.

### References

ALBERTS, D.S., GARCIA, D. & MASON-LIDDIL, N. (1991). Cisplatin in advanced cancer of the cervix: an update. *Semin. Oncol.*, **18**, 11–24.

ARUNDEL, C.M. & LEITH, J.T. (1987). Effects of nucleoside analogs and sodium butyrate on recovery from potentially lethal X ray damage in human colon tumor cells. *Int. J. Radiation Oncol. Biol. Phys.*, **13**, 593–601.

BOOTHBY, C.D., DANIEL, JILL, ADAM, SANDRA & DYSON, J.E.D. (1989). Use of a laser diffraction particle sizer for the measurement of mean diameter of multicellular tumour spheroids. *In Vitro*, **25**, 946–950.

CHESON, B.D., JASPERSE, D.M., CHUN, H.G. & FRIEDMAN, M.A. (1986). Differentiating agents in the treatment of human malignancies. *Cancer Treat. Rev.*, **13**, 129–145.

CONNORS, T.A. (1984). The chemotherapy of cervical cancer. In *Cancer of the Uterine Cervix, Biochemical and Clinical Aspects*, McBrien, D.C.H. & Slater, T.F. (eds), pp. 187–190. Academic Press: London.

DANIEL, P., BRAZIER, M., CERUTTI, J., PIERI, F., TARDIVEL, J., DESMET, G., BAILLET, J. & CHANY, C. (1989). Pharmacokinetic study of butyric acid administered in vivo as sodium and arginine butyrate salts. *Clin. Chim. Acta.*, **181**, 255–264.

DYSON, J.E.D., JOSLIN, C.A.F., QUIRKE, P. & BIRD, C.C. (1984*a*). Flow cytofluorometric analysis of serial biopsies of tumours of the uterine cervix. *Eur. J. Cancer Clin. Oncol.*, **20**, 1249–1259.

DYSON, J.E.D., QUIRKE, P., BIRD, C.C., MCLAUGHLIN, J.B. & SURREY, C.R. (1984*b*). Relationship between cell ploidy and glucocorticoid induced death in human lymphoid cell lines. *Br. J. Cancer*, **49**, 731–738.

DYSON, J.E.D., MCLAUGHLIN, J.B., SURREY, C.R., SIMMONS, D.M. & DANIEL, J. (1987). Effects of hyperthermia, irradiation and cytotoxic drugs on fluorescein isothiocyanate staining intensity for flow cytofluorometry. *Cytometry*, **8**, 26–34.

FRANKFURT, O.S. (1982). Intercellular adhesion in butyrate treated HeLa S3 cultures. *Exp. Cell Res.*, **139**, 257–263.

GERDES, J., LEMKE, H., BAISCH, H., WACKER, H.-H., SCHWAB, U. & STEIN, H. (1984). Cell cycle analysis of a cell proliferation-associated human nuclear antigen defined by the monoclonal antibody Ki-67. *J. Immunol.*, **133**, 1710–1715.

GERDES, J., LI, L., SCHLUETER, C., DUCHROW, M., WOHLENBERG, C., GERLACH, C., STAHMER, I., KLOTH, S., BRANDT, E. & FLAD, H. (1991). Immunobiochemical and molecular biologic characterization of the cell proliferation associated antigen that is defined by monoclonal antibody Ki67. *Am. J. Path.*, **138**, 867–873.

GLEESON, R.P., AYUB, M., WRIGHT, J.T., WOOD, C.B., HABIB, N.A., SOUTTER, W.P., SULLIVAN, M.H.F. & WHITE, J.O. (1990). Fatty acid control of growth of human cervical and endometrial cancer cells. *Br. J. Cancer*, **61**, 500–503.

KHOURY, G.G., BULMAN, A.S., JOSLIN, C.A.F. & ROTHWELL, R.I. (1991). Concomitant pelvic irradiation, 5 fluorouracil and mitomycin C in the treatment of advanced cervical carcinoma. *Br. J. Radiol.*, **64**, 252–260.

KRUH, J. (1982). Effect of sodium butyrate, a new pharmacological agent, on cells in culture. *Mol. Cell Biochem.*, **42**, 65–82.

LEITH, J.T., HALLOWS, K.T., ARUNDEL, C.M. & BLIVEN, S.F. (1988). Changes in X-ray sensitivity and glutathione content of human colon tumor cells after exposure to the differentiating inducing agent sodium butyrate. *Radiat. Res.*, **114**, 579–588.

MCGARVEY, T.W., SILBERMAN, S. & PERKSY, B. (1990). The effect of butyric acid and retinoic acid on invasion and experimental metastasis of murine melanoma cells. *Clin. Expl. Metastasis*, **8**, 433–448.

MILLER, A.A., KURSCHEL, E., OSIEKA, R. & SCHMIDT, C.G. (1987). Clinical pharmacology of sodium butyrate in patients with acute leukaemia. *Eur. J. Cancer Clin. Oncol.*, **23**, 1283–1287.

NORDENBERG, J., WASSERMAN, L., PELED, A., MALIK, Z., STENZEL, K.H. & NOVOGRODSKY, A. (1987). Biochemical and ultrastructural alterations accompany the anti-proliferative effect of butyrate on melanoma cells. *Br. J. Cancer*, **55**, 493–497.

NOVOGRODSKY, A., DVIR, A., RAVID, A., SHKOLNIK, T., STENZEL, K.H., RUBIN, A.L. & ZEIYOR, R. (1983). Effect of polar organic compounds on leukemic cells: butyrate induced partial remission of acute myelogenous leukemia in a child. *Cancer*, **51**, 9–14.

PLANCHON, P., RAUX, H., MAGNIEN, V., RONCO, G., VILLA, P., CREKIN, M. & BROUTY-BOYE, D. (1991). New stable butyrate derivates alter proliferation and differentiation in human mammary cells. *Int. J. Cancer*, **48**, 443–449.

PRASAD, K.N. (1980). Butyric acid: a small fatty acid with diverse biological functions. *Life Sci.*, **27**, 1351–1358.

SABIR, A.A., KHOURY, G.G., JOSLIN, C.A.F. & HEAD, C. (1989). Treatment of recurrent and metastatic carcinoma of cervix with chemotherapy: a comparison of low dose methotrexate with adriamycin and methotrexate. *Clin. Oncol.*, **1**, 70–74.

SPECTOR, A.A. & BURNS, C.P. (1987). Biological and therapeutic potential of membrane lipid modification in tumours. *Cancer Res.*, **47**, 4529–4537.

SPREMULI, E.N. & DEXTER, D.L. (1984). Polar solvents: a novel class of antineoplastic agents. *J. Clin. Oncol.*, **2**, 227–235.

SUTHERLAND, R.M. & DURAND, R.E. (1976). Radiation response of multicellular spheroids – an *in vitro* tumour model. *Curr. Top. Radiat. Res.*, **11**, 87–139.

WASSERMAN, L., BEERY, E., AVIRAM, R., LEVAVI, H., OVADIA, J., NOVOGRODSKY, A. & NORDENBERG, J. (1989). Sodium butyrate enhances the activities of membranal enzymes and increases drug sensitivity in a cell line from ascitic fluid of an ovarian carcinoma patient. *Eur. J. Cancer Clin. Oncol.*, **25**, 1765–1768.

WATSON, J.V., CHAMBERS, S.H. & SMITH, P.J. (1987). A pragmatic approach to the analysis of DNA histograms with a definable $G_1$ peak. *Cytometry*, **8**, 1–8.

WELANDER, C.E. & PARKER, R.L. Jr (1987). Human tumour clonogenic assay studies of cervical cancer. In *Cervix Cancer*, Surwit, E.A. & Albert, D.S. (eds), pp. 185–197. Martin Nijhoff: Boston, USA.


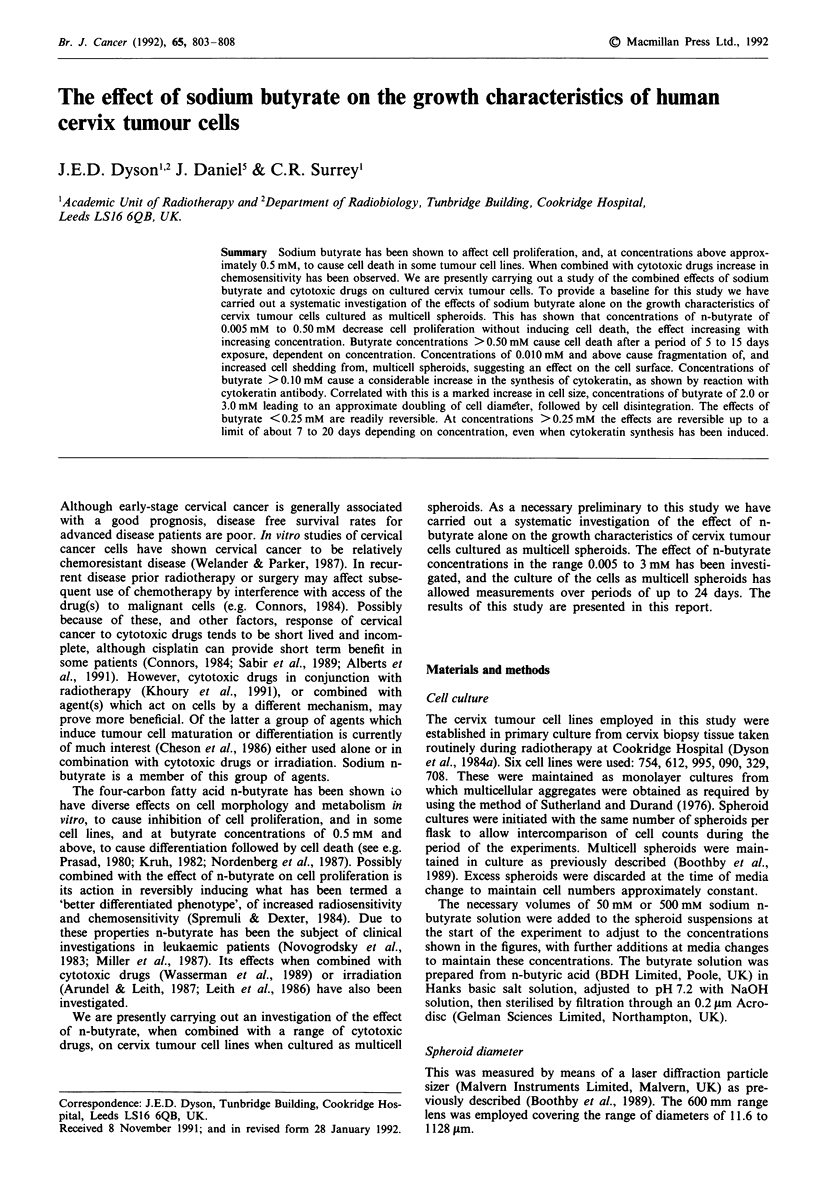

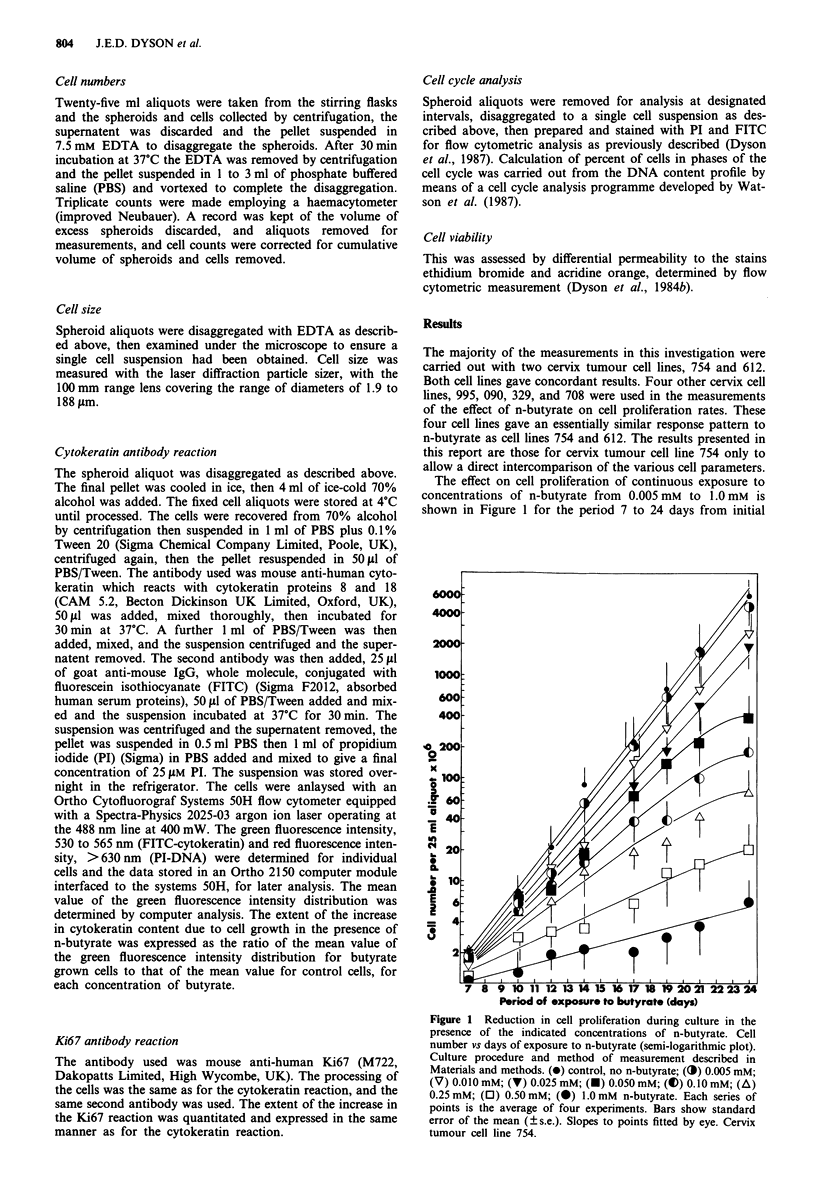

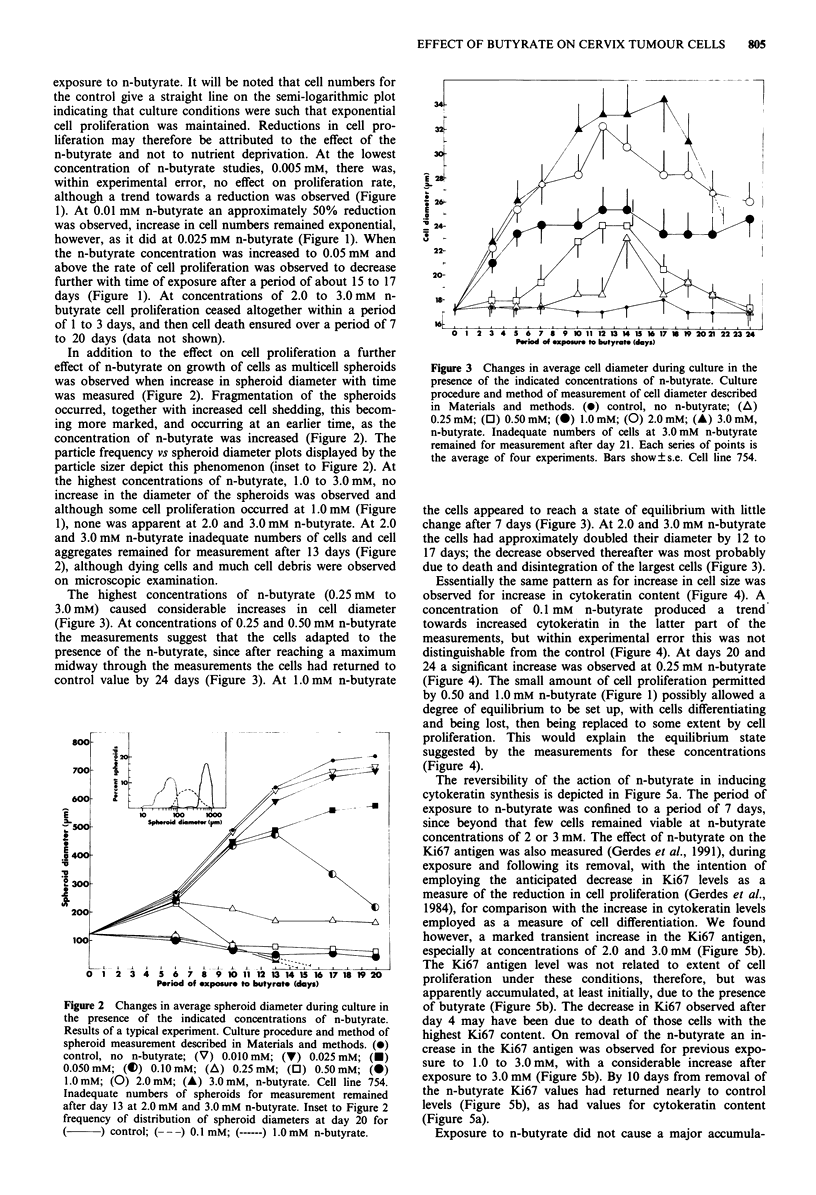

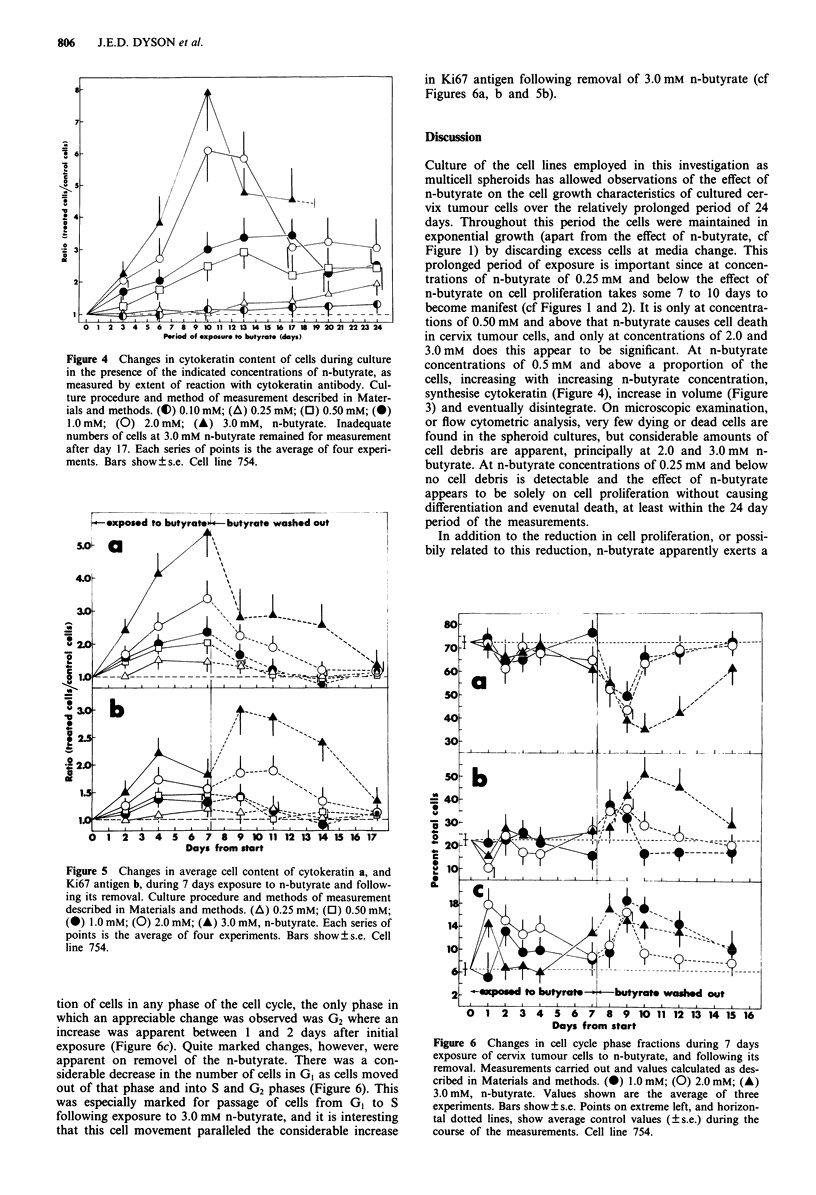

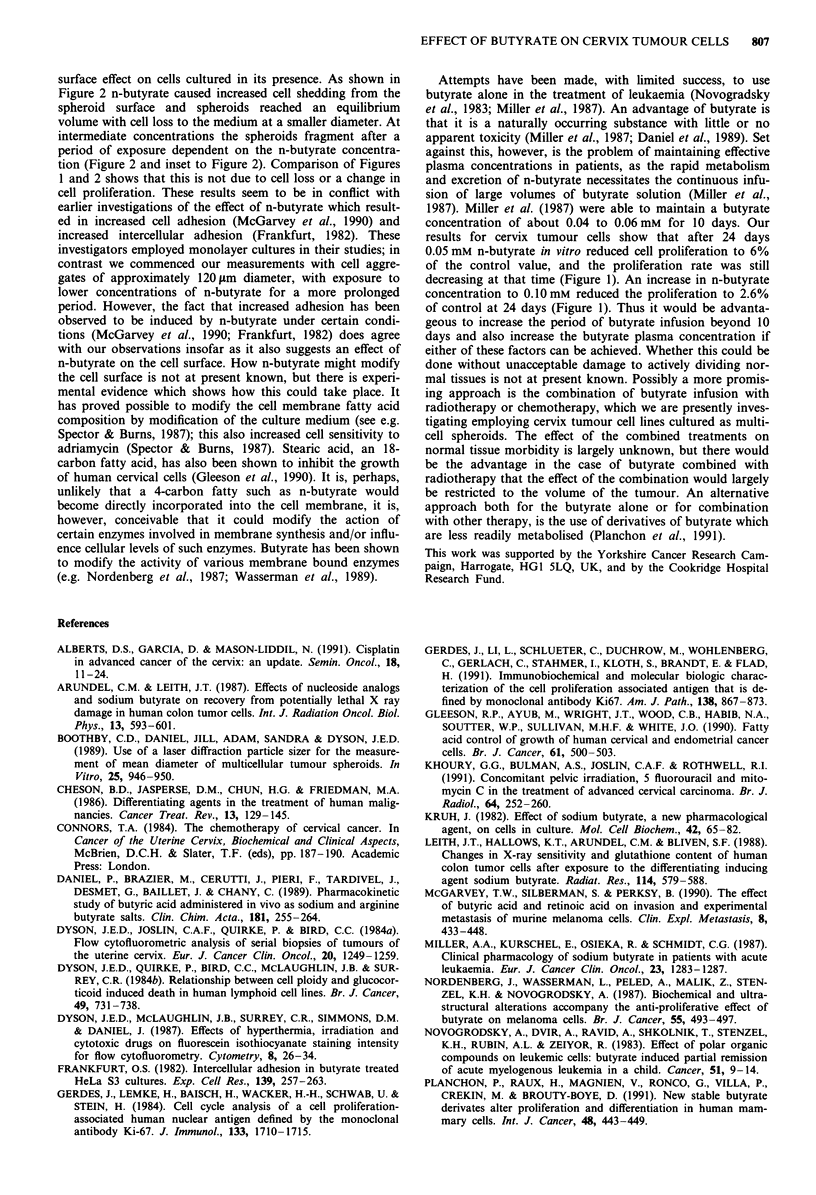

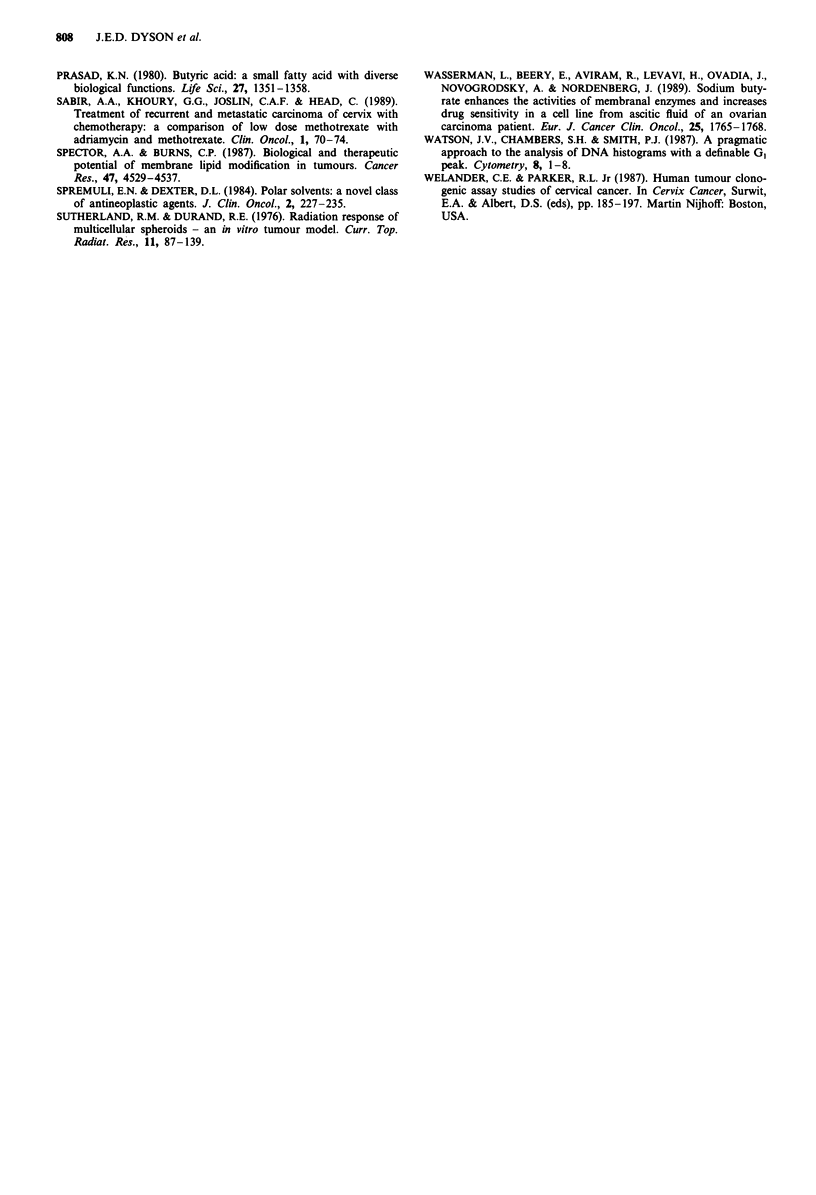


## References

[OCR_00598] Alberts D. S., Garcia D., Mason-Liddil N. (1991). Cisplatin in advanced cancer of the cervix: an update.. Semin Oncol.

[OCR_00603] Arundel C. M., Leith J. T. (1987). Effects of nucleoside analogs and sodium butyrate on recovery from potentially lethal X ray damage in human colon tumor cells.. Int J Radiat Oncol Biol Phys.

[OCR_00609] Boothby C. D., Daniel J., Adam S., Dyson J. E. (1989). Use of a laser diffraction particle sizer for the measurement of mean diameter of multicellular tumor spheroids.. In Vitro Cell Dev Biol.

[OCR_00615] Cheson B. D., Jasperse D. M., Chun H. G., Friedman M. A. (1986). Differentiating agents in the treatment of human malignancies.. Cancer Treat Rev.

[OCR_00626] Daniel P., Brazier M., Cerutti I., Pieri F., Tardivel I., Desmet G., Baillet J., Chany C. (1989). Pharmacokinetic study of butyric acid administered in vivo as sodium and arginine butyrate salts.. Clin Chim Acta.

[OCR_00632] Dyson J. E., Joslin C. A., Quirke P., Bird C. C. (1984). Flow cytofluorometric analysis of serial biopsies of tumours of the uterine cervix.. Eur J Cancer Clin Oncol.

[OCR_00642] Dyson J. E., McLaughlin J. B., Surrey C. R., Simmons D. M., Daniel J. (1987). Effects of hyperthermia, irradiation, and cytotoxic drugs on fluorescein isothiocyanate staining intensity for flow cytofluorometry.. Cytometry.

[OCR_00638] Dyson J. E., Quirke P., Bird C. C., McLaughlin J. B., Surrey C. R. (1984). Relationship between cell ploidy and glucocorticoid induced death in human lymphoid cell lines.. Br J Cancer.

[OCR_00648] Frankfurt O. S. (1982). Intercellular adhesion in butyrate-treated HeLa S3 cultures. Flow cytometric analysis.. Exp Cell Res.

[OCR_00652] Gerdes J., Lemke H., Baisch H., Wacker H. H., Schwab U., Stein H. (1984). Cell cycle analysis of a cell proliferation-associated human nuclear antigen defined by the monoclonal antibody Ki-67.. J Immunol.

[OCR_00658] Gerdes J., Li L., Schlueter C., Duchrow M., Wohlenberg C., Gerlach C., Stahmer I., Kloth S., Brandt E., Flad H. D. (1991). Immunobiochemical and molecular biologic characterization of the cell proliferation-associated nuclear antigen that is defined by monoclonal antibody Ki-67.. Am J Pathol.

[OCR_00664] Gleeson R. P., Ayub M., Wright J. T., Wood C. B., Habib N. A., Soutter W. P., Sullivan M. H., White J. O. (1990). Fatty acid control of growth of human cervical and endometrial cancer cells.. Br J Cancer.

[OCR_00670] Khoury G. G., Bulman A. S., Joslin C. A., Rothwell R. I. (1991). Concomitant pelvic irradiation, 5-fluorouracil and mitomycin C in the treatment of advanced cervical carcinoma.. Br J Radiol.

[OCR_00676] Kruh J. (1982). Effects of sodium butyrate, a new pharmacological agent, on cells in culture.. Mol Cell Biochem.

[OCR_00680] Leith J. T., Hallows K. T., Arundel C. M., Bliven S. F. (1988). Changes in X-ray sensitivity and glutathione content of human colon tumor cells after exposure to the differentiation-inducing agent sodium butyrate.. Radiat Res.

[OCR_00686] McGarvey T. W., Silberman S., Persky B. (1990). The effect of butyric acid and retinoic acid on invasion and experimental metastasis of murine melanoma cells.. Clin Exp Metastasis.

[OCR_00692] Miller A. A., Kurschel E., Osieka R., Schmidt C. G. (1987). Clinical pharmacology of sodium butyrate in patients with acute leukemia.. Eur J Cancer Clin Oncol.

[OCR_00699] Nordenberg J., Wasserman L., Peled A., Malik Z., Stenzel K. H., Novogrodsky A. (1987). Biochemical and ultrastructural alterations accompany the anti-proliferative effect of butyrate on melanoma cells.. Br J Cancer.

[OCR_00703] Novogrodsky A., Dvir A., Ravid A., Shkolnik T., Stenzel K. H., Rubin A. L., Zaizov R. (1983). Effect of polar organic compounds on leukemic cells. Butyrate-induced partial remission of acute myelogenous leukemia in a child.. Cancer.

[OCR_00709] Planchon P., Raux H., Magnien V., Ronco G., Villa P., Crépin M., Brouty-Boyé D. (1991). New stable butyrate derivatives alter proliferation and differentiation in human mammary cells.. Int J Cancer.

[OCR_00717] Prasad K. N. (1980). Butyric acid: a small fatty acid with diverse biological functions.. Life Sci.

[OCR_00721] Sabir A. A., Khoury G. G., Joslin C. A., Head C. (1989). Treatment of recurrent and metastatic carcinoma of cervix: a comparison of low dose methotrexate with adriamycin plus methotrexate.. Clin Oncol (R Coll Radiol).

[OCR_00727] Spector A. A., Burns C. P. (1987). Biological and therapeutic potential of membrane lipid modification in tumors.. Cancer Res.

[OCR_00732] Spremulli E. N., Dexter D. L. (1984). Polar solvents: a novel class of antineoplastic agents.. J Clin Oncol.

[OCR_00736] Sutherland R. M., Durand R. E. (1976). Radiation response of multicell spheroids--an in vitro tumour model.. Curr Top Radiat Res Q.

[OCR_00741] Wasserman L., Beery E., Aviram R., Levavi H., Ovadia J., Novogrodsky A., Nordenberg J. (1989). Sodium butyrate enhances the activities of membranal enzymes and increases drug sensitivity in a cell line from ascitic fluid of an ovarian carcinoma patient.. Eur J Cancer Clin Oncol.

[OCR_00747] Watson J. V., Chambers S. H., Smith P. J. (1987). A pragmatic approach to the analysis of DNA histograms with a definable G1 peak.. Cytometry.

